# Population history and genomic signatures for high-altitude adaptation in Tibetan pigs

**DOI:** 10.1186/1471-2164-15-834

**Published:** 2014-10-01

**Authors:** Huashui Ai, Bin Yang, Jing Li, Xianhua Xie, Hao Chen, Jun Ren

**Affiliations:** Key Laboratory for Animal Biotechnology of Jiangxi Province and the Ministry of Agriculture of China, Jiangxi Agricultural University, Nanchang, 330045 P. R China

**Keywords:** High-altitude adaptation, Genetic basis, Population history, Tibetan pigs

## Abstract

**Background:**

The Tibetan pig is one of domestic animals indigenous to the Qinghai-Tibet Plateau. Several geographically isolated pig populations are distributed throughout the Plateau. It remained an open question if these populations have experienced different demographic histories and have evolved independent adaptive loci for the harsh environment of the Plateau. To address these questions, we herein investigated ~ 40,000 genetic variants across the pig genome in a broad panel of 678 individuals from 5 Tibetan geographic populations and 34 lowland breeds.

**Results:**

Using a series of population genetic analyses, we show that Tibetan pig populations have marked genetic differentiations. Tibetan pigs appear to be 3 independent populations corresponding to the Tibetan, Gansu and Sichuan & Yunnan locations. Each population is more genetically similar to its geographic neighbors than to any of the other Tibetan populations. By applying a locus-specific branch length test, we identified both population-specific and -shared candidate genes under selection in Tibetan pigs. These genes, such as *PLA2G12A*, *RGCC*, *C9ORF3*, *GRIN2B*, *GRID1* and *EPAS1*, are involved in high-altitude physiology including angiogenesis, pulmonary hypertension, oxygen intake, defense response and erythropoiesis. A majority of these genes have not been implicated in previous studies of highlanders and high-altitude animals.

**Conclusion:**

Tibetan pig populations have experienced substantial genetic differentiation. Historically, Tibetan pigs likely had admixture with neighboring lowland breeds. During the long history of colonization in the Plateau, Tibetan pigs have developed a complex biological adaptation mechanism that could be different from that of Tibetans and other animals. Different Tibetan pig populations appear to have both distinct and convergent adaptive loci for the harsh environment of the Plateau.

**Electronic supplementary material:**

The online version of this article (doi:10.1186/1471-2164-15-834) contains supplementary material, which is available to authorized users.

## Background

The Qinghai-Tibet Plateau, known as the “roof of the world”, is the highest (mostly at 3,500 - 4,500 m) and largest (~2,500,000 km^2^) highland on the earth. The environmental condition of the plateau is characterized by the reduced oxygen availability, low ambient temperature, high ultraviolent radiation and amid climate. The unique ecological condition imposes severe physiological challenges on inhabits in the high-altitude region. Native inhabitants like Tibetans have evolved the adaptive mechanism to address the harsh environment during the long history of colonization. Today, a number of researchers have identified particular physiological traits favoring local adaption in Tibetans. The adaptive traits include increased nitric oxide level, elevated resting ventilation, reduced pulmonary vasoconstrictor response and low hemoglobin concentration compared with acclimated lowlanders [1–3].

Although the physiological traits in response to high-altitude environments are relatively well characterized, an understanding of the molecular basis underlying these traits has significantly lagged behind. Recently, population genomics offers an effective approach to characterize the adaptive mechanism of Tibetans and other colonists in the Qinghai-Tibet Plateau. One common approach is involved in genotyping a large number of loci across the genome on divergently differentiated populations, and searching “outliers” (candidate targets of selection) in the extreme tail of the empirical distribution of statistics like F_ST_ [4, 5] and locus-specific branch length (LSBL) values [6–8]. To date, a series of genomic scan researches have highlighted more than a dozen of candidate genes subject to natural selection in Tibetans [4, 5, 8–11]. Two major candidate genes (*EGLN1* and *EPAS1*) in the hypoxia-inducible factor (HIF) pathway have been concordantly shown to carry the adaptive mutations [12]. Both *EPAS1* [9] and *EGLN1* [10, 11] variants are associated with the adaptively low hemoglobin level of Tibetans relative to acclimatized lowlanders. Recently, a non-synonymous mutation in the *EGLN1* gene is suggested to be the causal variant for local adaption in Tibetans [11]. Intriguingly, Tibetans show distinct adaptive mechanism as compared to other highland populations including Ethiopians [7, 13] and Andeans [6], in which different variants in *EGLN1* and different selection-nominated candidate genes like *BHLHE41* have been identified. In non-human species indigenous to the Qinghai-Tibet plateau, the draft genomes of the Tibetan antelope [14] and Yak [15] have been recently generated. Adaptive signals have been detected in genes associated with oxygen transmission, energy metabolism and DNA repair in the two species, improving our understanding of the genetic mechanisms of high-altitude adaptation in highland animals.

**Figure 1:**
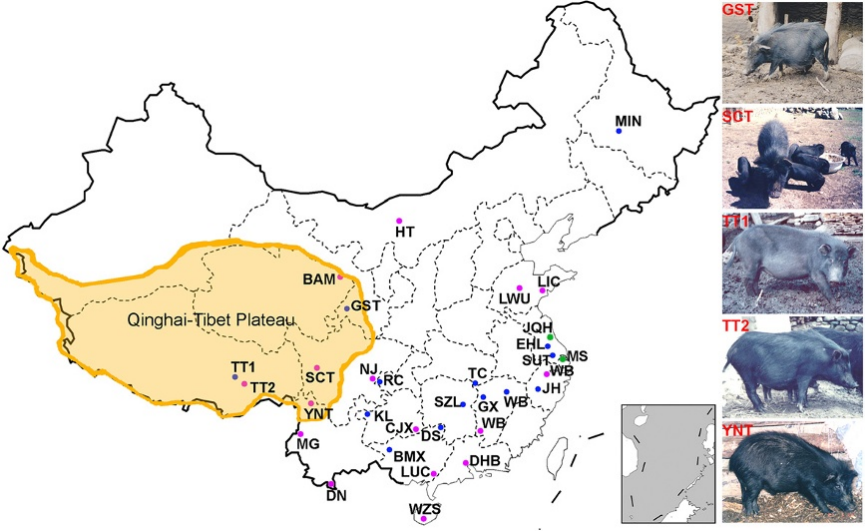
**Map of sample locations in the present study.** Our samples were collected from 5 Tibetan pig geographic populations and 25 Chinese lowland breeds. Populations tested in this study are highlighted in pink. Our previously reported and online available breeds are indicated in blue and green, respectively. Phenotypes of the 5 Tibetan pig geographic populations are shown in the left panel. BAM, Bamei; BMX, Bama Xiang; CJX, Congjiang Xiang; DHB, Dahuabai; DN, Diannanxiaoer; DS, Dongshan; EHL, Erhualian; GST, Tibetan (Gansu); GX, Ganxi; HT, Hetaodaer; JH, Jinhua; JQH, Jiangquhai; KL, Kele; LIC, Lichahei; LWU, Laiwu; LUC, Luchuan; MG, Mingguangxiaoer; MIN, Min; MS, Meishan; NJ, Neijiang; RC, Rongchang; SCT, Tibetan (Sichuan); SUT, Sutai; SZL, Shaziling; TC, Tongcheng; TT1, Tibetan (Gongbujiangda); TT2, Tibetan (Milin); WB, Chinese wild boars; WZS, Wuzhishan; YNT, Tibetan (Yunnan).

The Tibetan pig, one of Tibetans’ domestic animals, is originally distributed in farming and farming-pastoral regions at altitudes of 2,900 - 4,300 m in the Qinghai-Tibetan Plateau [16]. Like Tibetans and other colonists in the Plateau, Tibetan pigs have several key adaptive features for coping with the harsh environment at high altitude [16]. First, they have black skin and hair with long and dense bristle that protect them from high solar radiation and cold ambient temperature in winter (Figure 1). Second, their well-developed heart and lung may be required for the increased rate of blood flow and oxygen transportation to tissues in response to hypoxia. Third, they have developed a blunted erythropoietic response to high-altitude hypoxia by exhibiting lower than expected hemoglobin concentrations relative to their lowland counterparts and acclimatized lowland pigs [17]. This feature, in contrast to Tibetan yak, goat and sheep that show elevated hemoglobin levels at high altitude, is a crucial protection mechanism for excessive erythrocytosis (a classic feature of chronic mountain sickness [18]) in Tibetans [1–3]. Forth, their alertness and agility enable them to survive in the grazing condition.

To elucidate the genetic basis of the altitude phenotypes in Tibetan pigs, we have previously searched for population-differentiated SNPs across the genome between two Tibetan pig populations and 16 lowland breeds using the Illumina 60 K SNP data. We identified several candidate genes that might play a role in high-altitude adaptation in Tibetan pigs [19]. More recently, Li et al. [20] characterize fast evolved genes in Tibetan pigs and highlight a set of genes that may contribute to high-altitude adaptation using whole-genome sequence data. The two studies treated all Tibetan pigs as a single population. However, the Qinghai-Tibet Plateau stretches across a vast region. Several geographically isolated populations of the Tibetan pig are currently living in the region, including Sichuan, Yunnan, Gansu and Tibet populations [16]. Environmental factors including temperature, humidity, precipitation and vegetation are variable across the hypoxic and high-ultraviolet plateau [16]. Therefore, different Tibetan geographical populations may have experienced independent adaptive mechanism for the harsh environment. Besides, it remains an open question whether Tibetan pig populations have experienced differentiation and subdivisions during the long period of their colonization in the Plateau.

Here we genotyped a broad panel of 678 pigs from 5 Tibetan regional populations and 34 lowland breeds using high-density SNP genotyping arrays. By applying population genetic and genomic approaches, we characterize the genetic landscape for Tibetan pigs and reveal both population-shared and -specific candidate genes contributing to local adaptations in the 5 Tibetan populations. Our findings provide novel insights into evolutionary history of Tibetan pigs and the genetic architecture of high-altitude adaptation in highland animals and their owners.

## Results

### Population structure and evolution history of Tibetan pigs

To investigate population structure of Tibetan pigs, we first constructed a neighbor-joining (NJ) tree based on genome-wide allele sharing of the 678 pigs from 5 Tibetan populations, 28 Chinese lowland breeds (Figure 1, Additional file 1: Table S1) and 6 Western breeds (Additional file 1: Table S1). A notable feature of the NJ tree is the high concordance with which individuals cluster to their population origin. The topological tree (Additional file 2: Figure S1) clearly illustrates that all individuals from the same population or breed gather together, and Western pigs form a cluster separating from Chinese indigenous pigs. The genetic relationships between Chinese breeds are strikingly concordant with their geographic locations. For instance, Erhualian, Meishan, Jiangquhai, Jinhua, Tongcheng, Ganxi and Shaziling from the middle-lower belt of Yangtze River defined a separate grouping, while breeds from South China and Southwest China including Luchuan, Wuzhishan, Bamaxiang, Dahuabai, Congjiang Xiang and Diannan pigs appeared as a closely related cluster. Intriguingly, five Tibetan regional populations did not form a separate cluster. Instead, Tibetan pigs from Gansu province were more closely related to North China breeds like Bamei and Hetao than to any of the other Tibetan populations. Moreover, Tibetan pigs from Sichuan and Yunnan provinces were grouped together with their geographic neighbors, Rongchang and Neijiang. The two populations (Gongbujiangda and Milin) in the Tibet Autonomous Region clustered in an independent clade.

To better understand the population structure of Tibetan pigs, we further performed a principal component analysis (PCA) using a subset of 14,202 SNPs with low linkage disequilibrium (LD) extents (*r*^2^ < 0.3) in all tested pigs. The PCA plot (Additional file 3: Figures S2) shows the differentiation pattern between Chinese and Western pigs that is highly consistent the NJ tree results. The differentiation patterns of the five Tibetan regional populations resemble the NJ results. The Tibetan pigs from Yunnan and Sichuan provinces exhibit strong genetic affinity to their geographic neighbors including Neijing and Rongchang, whereas the Gansu Tibetan population cluster near their neighbors, Bamei and Hetao pigs. The Gongbujiang and Milin populations are more genetically similar to each other than to any of the other Tibetan populations.

To assess evolutionary origin and historical admixture patterns of Tibetan pigs in a context of worldwide populations, we further conducted a Bayesian ancestry inference analysis using the program ADMIXTURE (Additional file 4: Figure S3). Within Chinese pigs, from K = 3 to K = 7, variable fractions of Chinese wild ancestry were evidenced. At K = 7 and 8, five ancestry fractions were detected in Chinese breeds, including the ancestors of Gongbujiangda Tibetan, Congjiang Xiang, Luchan, Jinhua and Erhualian pigs. When we focused on Tibetan pigs, the Gansu Tibetan population was derived from two ancestry fractions of Gongbujiangda Tibetan (70%) and Erhualian (30%) pigs, and its ancestry structure was similar to their geographic neighbors: Bamei and Hetao pigs. The ancestry structures of Tibetan pigs from Sichuan and Yunnan were nearly identical, which consist of 75% of Gongbujiangda Tibetan, 10% of Luchuan and 15% of Erhualian ancestry fractions and resemble the structures of their lowland neighbors including Neijing, Rongchang and Mingguang pigs.

**Figure 2:**
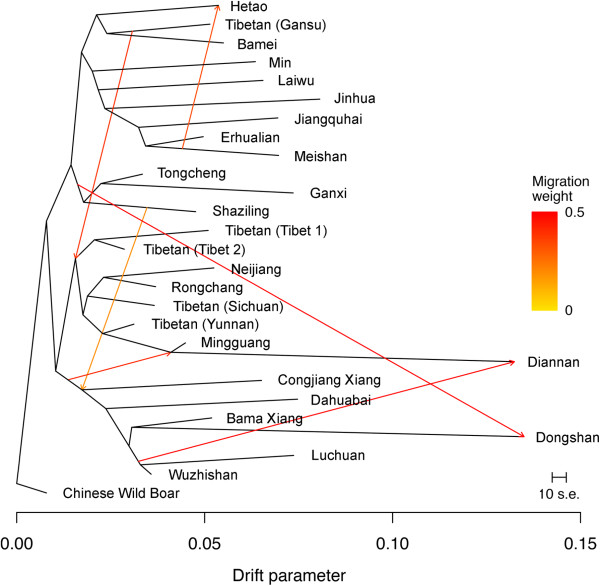
**Population split and historical mixture for Tibetan pigs in a context of Chinese diverse breeds.** Arrows indicate migration events among Chinese indigenous breeds. A spectrum of heat colors indicates different migration weights at the migration event.

To further infer population splits and mixtures of Tibetan pigs, we used a recently developed approach, Treemix [21], to construct a maximum-likelihood tree of the 5 Tibetan populations, 21 Chinese lowland breeds and 1 Chinese wild boar population. A close examination of residuals from the inferred tree without migration events (Additional file 5: Figure S4) revealed that 6 pairs of populations were apparently not compatible with the best-fit tree, suggestive of gene flow events. Indeed, the tree model without migration events only explained 89.9% of the variance in the relatedness between populations. We sequentially added migration events to the maximum-likelihood tree (Figure 2). The new tree model allowing 6 major migration events explained higher percentage (96.0%) of the variance in the relatedness between populations. In the inferred graph (Figure 2), the regional populations from Tibet, Yunnan and Sichuan provinces were grouped into one of the two major groupings, whereas the Gansu Tibetan population clusters with the other major grouping consisting of Bamei and Hetao pigs. An ancestral edge further divided Tibet and Yunnan/Sichuan populations into different subgroups. Again, Yunan and Sichuan Tibetan populations clustered with their geographic neighbors Neijiang and Rongchang. Of the six migration events, the strongest signal suggests a genetic contribution from the ancestry of Tongcheng, Shaziling and Ganxi breeds to Dongshan pigs (migration weight (w) = 49.9%). Another visually apparent event is a gene flow from the ancestry of Wuzhishan and Luchuan pigs into Diannan pigs (w = 49.2%). We also inferred a significant admixture event from Meishan to Hetao individuals (w = 33.8%). For Tibetan pigs, we found a gene flow event from the early Gansu Tibetan population into the ancestry of the other four Tibetan populations with a migration weight of 38.8%.

Taken together, we conclude that Tibetan pig geographic populations have experienced substantial genetic differentiation and population admixture. During the formation of current populations, Tibetan pigs were likely influenced by their geographic neighbors. Tibetan pigs are thus not a single breed, which appear to be 3 independent populations corresponding to the Tibetan, Gansu, and Sichuan & Yunnan locations (thereafter namely SCYN).

### Population-specific genomic signatures of selection in Tibetan pigs

According to the above-mentioned population genetic analyses, we divided Tibetan pigs into three independent populations: Tibet, Gansu and SCYN. To identify population-specific loci under positive selection in Tibetan pigs, we calculated the LSBL value for each of the 41,495 informative SNPs along the genome using a three-group contrasting model: one Tibetan population, one Chinese lowland group and the other two Tibetan populations (See Methods). The three-group test identified SNP outliers that had highly differentiated allele frequencies in one Tibetan population relative to the other two groups. These outlier SNPs are candidate loci (or to be in linkage disequilibrium with selected variants) under selection for adaptation to high-altitude hypoxia.

**Figure 3:**
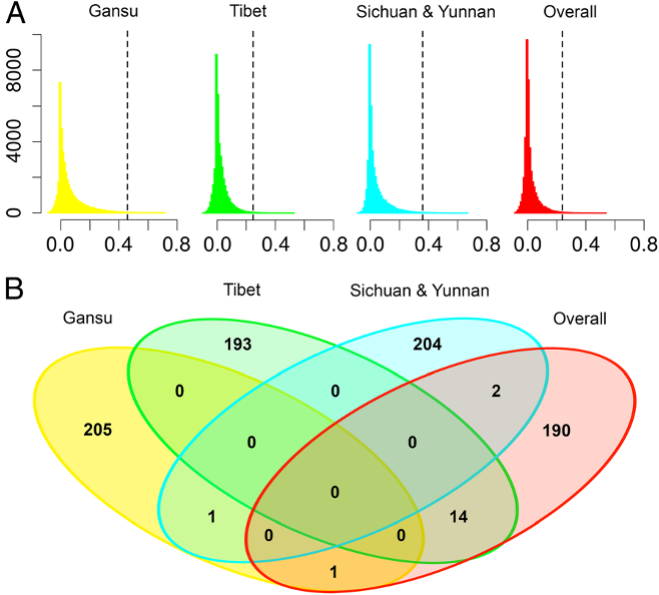
**LSBL analysis identifies candidate loci under selection for high-altitude adaptation in Tibetan pigs. (A)** Histograms of distribution of locus-specific branch length (LSBL) values in each and all Tibetan populations are depicted. LSBL values are shown in the x-axis and the number of individuals in the y-axis. Dashed grey lines indicate the significant thresholds: 0.5% of the empirical distribution. **(B)** Venn diagram shows shared and distinct candidate genes under selection in three Tibetan pig populations from Gansu, Tibet and Sichuan & Yunnan provinces. Numbers indicating how many outlier SNPs belong to each of Tibetan populations are shown in the panel.

**Figure 4:**
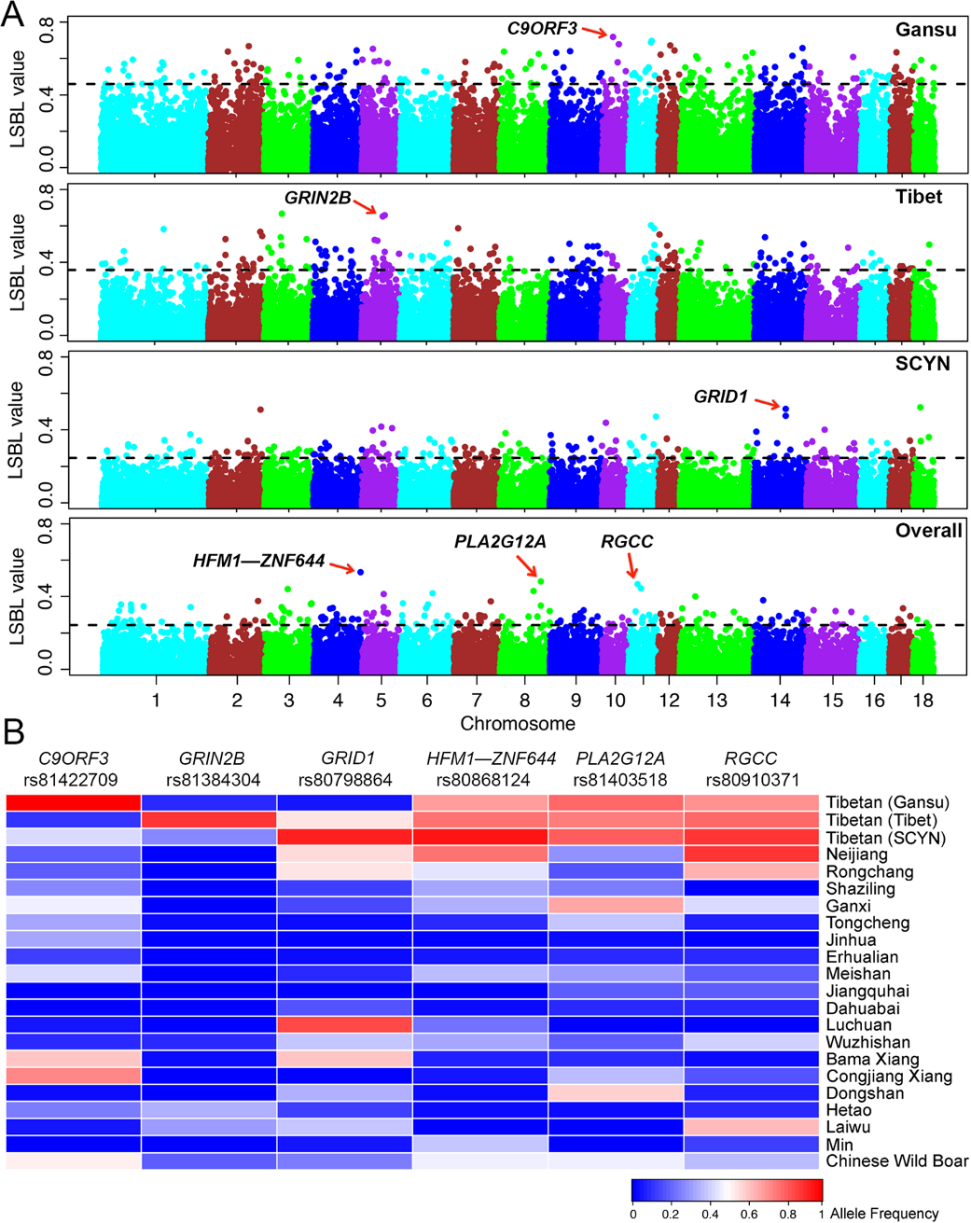
**Genomic signatures of selection in each and all Tibetan pig populations. (A)** Genome-wide distribution of LSBL values. From top to bottom panels, LSBL outliers are shown for each Tibetan pig population from Gansu, Tibet and Sichuan & Yunnan (SCYN) provinces as well as all Tibetan pigs. The chromosomes are plotted along the x-axis, and LSBL values are plotted along the y-axis. Chromosomes are indicated by different colors, and the threshold indicating signature of selection is denoted with a dashed grey line. The strongest candidate genes corresponding to the top SNP outliers are indicated by red arrows in each panel, and the gene names are labeled above the arrows. Two flanking genes (*HFM1* and *ZNF644*) are shown for one intergenic SNP. **(B)** A heat map of allele frequencies at the top SNP loci for each and overall of the tested populations. A spectrum of heat map colors indicates different allele frequencies at these loci.

We identified a total of 207 SNP outliers (0.5% of empirical LSBL distribution), corresponding to 140, 129 and 124 candidate genes (50 kb up- and downstream of each SNP outliers) in Gansu, Tibet and SCYN populations, respectively (Additional file 6: Table S2). Few common signals were found between the three Tibetan populations (Figure 3, Additional file 7: Figure S5). *C9ORF3*, *GRIN2B* and *GRID1*, three functionally plausible genes (See Discussion), exhibited the most significant signals of selection in Gansu, Tibet and SCYN populations, respectively (Figure 4A). These SNPs showed a marked allele frequency difference between one Tibetan population and the other Tibetan and lowland populations (Figure 4B).

We sequentially performed Gene Ontology (GO) and Kyoto Encyclopedia of Genes and Genomes (KEGG) pathway enrichment analyses on highlighted candidate genes in each Tibetan pig population. GO analysis identified 27, 15 and 17 overrepresented genes in Gansu, Tibet and SCYN populations, respectively (Table 1). These genes are involved in several biological processes that likely play a role in local adaptation of Tibetan pigs, such as neuron-neuron synaptic transmission (*GRIA1*, *GRIA4*, *GRM4*, *GRM5*, *HTR2A* and *VDAC1*, *P*_cor_ = 0.018), detection of mechanical stimulus involved in sensory perception (*GRIN2B*, *ITGA2* and *PCDH15*, *P*_cor_ = 0.029), artery development (*CHD7*, *FOXS1*, *GLI3*, and *MYLK2*, *P*_cor_ = 0.041), positive regulation of smooth muscle cell proliferation (*ID2* and *ITGA2*, *P*_cor_ = 0.041) and glutamate receptor signaling pathway (*GRIK2*, *HOMER2*, *NRXN1* and *PLCB1*, *P*_cor_ = 0.042). We also identified 4 and 3 KEGG pathways in Gansu and Tibet populations (Table 1) that were overrepresented after correction for multiple testing: basal cell carcinoma (*P*_cor_ = 0.031), circadian entrainment (*P*_cor_ = 0.033), nicotine addiction (*P*_cor_ = 0.043) and adherens junction (*P*_cor_ = 0.045) pathways in the Gansu population, and propanoate metabolism (*P*_cor_ = 0.004), sulfur metabolism (*P*_cor_ = 0.004) and DNA replication (*P*_cor_ = 0.024) in the Tibet population.

**Table 1:** **GO terms and KEGG pathways enriched with candidate genes for high-altitude adaptation in Tibetan pigs** ^**a**^

Population	ID	Term	***P***-value	Associated gene
	GO			
Gansu	GO:0030011	Maintenance of cell polarity	0.001	*ANK1*, *ANKH*, *DST*
	GO:0007270	Neuron-neuron synaptic transmission	0.018	*GRIA1*, *GRIA4*, *GRM4*, *GRM5*, *HTR2A*, *VDAC1*
	GO:0030282	Bone mineralization	0.028	*ANKH*, *BMP2*, *CLEC3B*, *LTF*, *TCF7L2*
	GO:0032663	Regulation of interleukin-2 production	0.041	*MALT1*, *PDE4D*
	GO:0048566	Embryonic digestive tract development	0.041	*GLI3*, *TCF7L2*
	GO:0060840	Artery development	0.041	*CHD7*, *FOXS1*, *GLI3*, *MYLK2*
	GO:0018345	Protein palmitoylation	0.042	*HHAT*, *ZDHHC3*, *ZDHHC7*
	GO:0016339	Calcium-dependent cell-cell adhesion	0.048	*CDH13*, *CDH17*, *CDH19*
Tibet	GO:0050974	Detection of mechanical stimulus involved in sensory perception	0.029	*GRIN2B*, *ITGA2*, *PCDH15*
	GO:0048661	Positive regulation of smooth muscle cell proliferation	0.041	*ID2*, *ITGA2*
	GO:0060079	Regulation of excitatory postsynaptic membrane potential	0.041	*GRIN2B*, *SEZ6*
	GO:0010922	Positive regulation of phosphatase activity	0.042	*ITGA2*, *MAGI2*, *PLEK*
	GO:0031532	Actin cytoskeleton reorganization	0.049	*ABL1*, *CSF1R*, *PARVA*, *PLEK*
Sichuan & Yunnan	GO:0097091	Synaptic vesicle clustering	0.010	*NRXN1*, *SYNDIG1*
	GO:0072337	Modified amino acid transport	0.024	*FOLR1*, *FOLR2*, *SLC1A4*
	GO:0072520	Seminiferous tubule development	0.034	*ING2*, *SPINK2*
	GO:0021575	Hindbrain morphogenesis	0.039	*FAIM2*, *NRXN1*
	GO:0060079	Regulation of excitatory postsynaptic membrane potential	0.039	*GRIK2*, *NRXN1*
	GO:1902930	Regulation of alcohol biosynthetic process	0.039	*APOB*, *TWIST1*
	GO:0017158	Regulation of calcium ion-dependent exocytosis	0.042	*B4GALT1*, *PLCB1*, *SYT1*
	GO:0006208	Pyrimidine nucleobase catabolic process	0.042	*DPYS*, *DPYSL3*
	GO:0007215	Glutamate receptor signaling pathway	0.042	*GRIK2*, *HOMER2*, *NRXN1*, *PLCB1*
Overall	GO:0010712	Regulation of collagen metabolic process	0.002	*CST3*, *ITGA2*, *PDGFRB*, *RGCC*
	GO:0051567	Histone H3-K9 methylation	0.018	*DNMT3B*, *KDM1A*, *PRDM5*
	GO:0050927	Positive regulation of positive chemotaxis	0.033	*CDH13*, *CNTN1*, *ITGA2*
	GO:0032465	Regulation of cytokinesis	0.038	*PDZD2*, *TEX14*
	GO:0019626	Short-chain fatty acid catabolic process	0.043	*MUT*, *PCCA*
	GO:0050966	Detection of mechanical stimulus involved in sensory perception of pain	0.043	*GRIN2B*, *ITGA2*
	GO:0002209	Behavioral defense response	0.045	*GRIN2B*, *NR2E1*, *VDAC1*
	KEGG			
Gansu	KEGG:05217	Basal cell carcinoma	0.031	*BMP2*, *GLI3*, *TCF7L2*
	KEGG:04713	Circadian entrainment	0.033	*ADCY10*, *GRIA1*, *GRIA4*, *RPS6KA5*
	KEGG:05033	Nicotine addiction	0.043	*GRIA1*, *GRIA4*
	KEGG:04520	Adherens junction	0.045	*NLK*, *PTPRB*, *TCF7L2*
Tibet	KEGG:00640	Propanoate metabolism	0.004	*ACACB*, *PCCA*, *SUCLG2*
	KEGG:00920	Sulfur metabolism	0.004	*IMPAD1*, *PAPSS2*
	KEGG:03030	DNA replication	0.024	*PRIM2*, *RPA1*
Overall	KEGG:00280	Valine, leucine and isoleucine degradation	0.036	*ACADSB*, *MUT*, *PCCA*
	KEGG:04975	Fat digestion and absorption	0.042	*APOB*, *PLA2G12A*
	KEGG:05014	Amyotrophic lateral sclerosis (ALS)	0.046	*DAXX*, *GRIN2B*, *MAP2K6*

^a^ *P*-values after Bonferroni correction for multiple testing. Overall indicates all Tibetan pig populations.

### Population-shared genomic signatures in all Tibetan pigs

To test if Tibetan pig populations have common adaptive loci for the Plateau, we also looked across the genome to identity signals of selection using the three-group test model with all Tibetan pigs as one group and two Chinese lowland groups (See Methods). A total of 207 SNP outliers were visualized on the top 0.5% of the empirical distribution, corresponding to 122 genes (Additional file 6: Table S2). The LSBL value plots and allele distribution patterns of the top three loci are depicted in Figure 4. Each top locus shows highly differentiation pattern between Tibetan pigs and Chinese lowland animals. For instance, at the top of the list is an intergenic SNP between *HFM1* and *ZNF644* (rs80868124, LSBL value = 0.535). Allele C at the SNP is predominantly presented in Tibetan pigs with average frequency of 0.816 whereas is much rarer in Chinese lowland breeds at an average frequency of 0.207 except for Neijiang (0.750), a breed known for its well adaptability to diverse environmental conditions [22]. The biological role of *HFM1* and *ZNF644* in response to hypoxia has not been established and warrants further investigations. The second strongest LSBL SNP (rs81403518) is located at 12 kb upstream of the *PLA2G12A* gene, a biologically plausible gene (see Discussion). The third strongest outlier is located at ~17 kb downstream of a hypoxia-inducible gene: *RGCC*.

GO analysis identified 16 overrepresented genes that are involved in the regulation of collagen metabolic process (*CST3*, *ITGA2*, *PDGFRB* and *RGCC*, *P*_cor_ = 0.002), histone H3-K9 methylation (*DNMT3B*, *KDM1A* and *PRDM5*, *P*_cor_ = 0.018), positive regulation of positive chemotaxis (*CDH13*, *CNTN1* and *ITGA2*, *P*_cor_ = 0.033), regulation of cytokinesis (*PDZD2* and *TEX14*, *P*_cor_ = 0.038), fatty acid catabolism (*MUT* and *PCCA*, *P*_cor_ = 0.043), detection of mechanical stimulus involved insensory perception of pain (*GRIN2B* and *ITGA2*, *P*_cor_ = 0.043), and behavioral defense response (*GRIN2B*, *NR2E1* and *VDAC1*, *P*_cor_ = 0.045) (Table 1). Meanwhile, we found 3 KEGG pathwaysthat were overrepresented after correction for multiple testing: valine, leucine and isoleucine degradation (*P*_cor_ = 0.036), fat digestion and absorption (*P*_cor_ = 0.042) and amyotrophic lateral sclerosis (*P*_cor_ = 0.046) (Table 1). KEGG:04975 (fat digestion and absorption) is particular interesting as it may reflect gene selection for fully utilizing fat as an energy source in case of food shortage, a critical ecological factor restricting the viability of highland animals.

### Signatures of selection in three well-characterized hypoxia genes

*EGLN1* and *EPAS1*, two critical regulators in the HIF pathway, have been repeatedly identified as targets of selection for high-altitude adaptation in Tibetans (reviewed in [12]). *ADAM17* is the most prominent locus showing signal of positive selection in the Tibetan yak [15]. We, therefore, are interested in whether any of the three genes has experienced convergent selection in Tibetan pigs. As no SNP on the current porcine 60 K chip is located around the three genomic regions, we sequentially genotyped 56 SNPs at an average density of 1 SNP/5.4 kb covering the three genes on a panel of 324 individuals including 84 Tibetan pigs and 240 lowland animals. The resulting genotype data were merged into the 60 K SNP dataset. We then performed the LSBL analysis on the 324 individuals using a common subset of informative SNPs. No significant selection signal was detected at both *EGLN1* and *ADAM17* loci (Additional file 8: Figure S6). Two of 22 SNPs around the *EPAS1* gene appeared to be population-shared outliers surpassing the significance threshold (LSBL value = 0.301, corresponding to the top 0.5% of empirical distribution). The two SNPs showed apparently different variation patterns between Tibetan pigs and low-altitude individuals (Additional file 8: Figure S6).

### Comparison of our findings with previous reports

**Figure 5:**
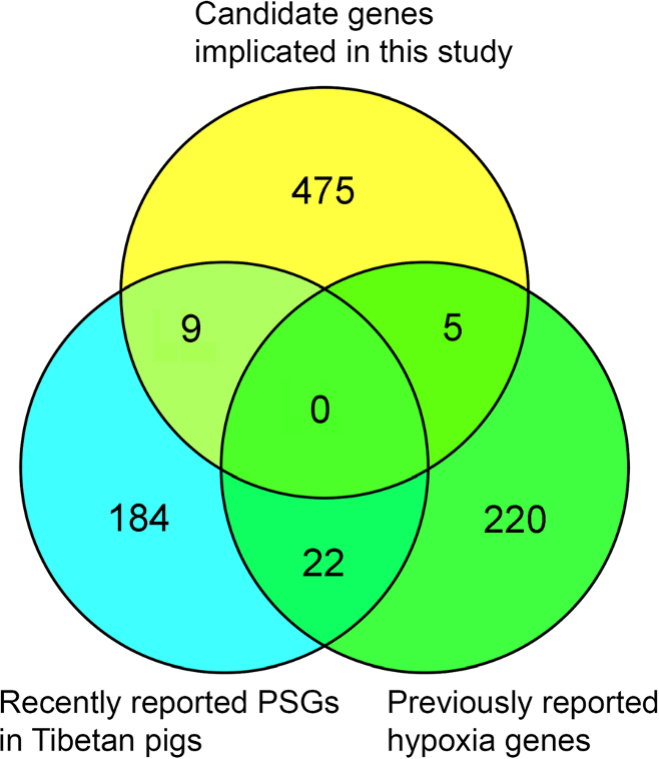
**Comparison of our highlighted candidate genes with previous reports.** A venn diagram showing shared and distinct candidate genes for high-altitude adaptation between our findings, the 247 previously reported hypoxia genes [14] and 215 positively selected genes (PSGs) recently identified in the Tibetan wild boars [20].

As shown in Figure 5, a majority of candidate genes implicated in the current study are distinct from those hypoxia genes identified in Tibetans [4–9], other highlanders [11–13] and Tibetan antelope [14] and yak [15]. We argue that Tibetan pigs may have evolved a different biological adaptation mechanism. Even compared with the recent findings of hypoxia-related genes in Tibetan pigs based on the whole-genome sequence data [19], most of candidate genes including those ranking in the top list of the current study (such as *PLA2G12A*, *RGCC*, *C9ORF3*, *GRIN2B* and *GRID1*) are reported for the first time.

## Discussion

### Population history of Tibetan pigs

The formation of Tibetan present-day pig populations was likely influenced by the migration of their owners: Tibetans. Today, both archaeological and genetic data support an ancient initial colonization of modern humans in the early Upper Paleolithic before the last glacial maximum (22,000 – 18,000 years ago) and a recent population expansion and gene flow from outside the highland region in the early Neolithic (10,000 – 7,000 years ago) [23]. The recent migration events are believed to bring agriculture into the Himalayan region, leading to the establishment of farming and yak pastoralism on the Plateau [24]. Since then, the trade between the Tibet and Southeastern/Northeastern China lasted for thousands of years through three major routines: the Tangbo Ancient Road and the Tea-horse Ancient Road including the Yunnan-Tibet and the Sichuan-Tibet routes. The Tangbo Ancient Road extends from Chang’an (the ancient capital of the Chinese Empire) to Lhasa. Historically, it was a very important trade route linking the Upper Yellow River region and the Tibet especially during the Dang Dynasty (618 – 907 A.D.). The Tea-horse Ancient Road played a crucial role in communication and exchange between the residents of Yunnan, Sichuan and Tibet. It had been flourishing for over a century until the end of World War II. The long-standing trade likely brought along human-mediated dispersal of lowland pigs in Southwestern and Northwestern China into the Tibetan Plateau. Genetic data from mitochondria DNA support a local domestication origin of Tibetan pigs [25]. However, there were multiple migrations of Tibetan ancestors at different times from different places and ample trade exchanges between the Tibet and the outside regions. This leads us to assume that the genetic makeup of current Tibetan pig populations may be influenced by their lowland neighbors, leading to population subdivisions of Tibetan pigs. The hypothesis is supported by our following observations. (1) In the PCA results, PC2 axis separated Tibetan pig populations into different groupings corresponding to their geographic locations. (2) In the NJ phylogenetic tree, the Gansu Tibetan population defined an independent branch with its neighbors including the Bamei pig in Qinghai and the Hetao pig in Inner Mongolia. The Sichuan and Yunnan Tibetan populations formed another cluster together with two lowland neighbors (Neijiang and Rongchang) in the Sichuan Basin. The two local populations from the Tibet appeared to be a distinct clade. (3) The Treemix analysis showed the evolutionary splits between the Tibetan populations. The Gansu Tibetan population was assigned to one ancient major group separating from the other Tibetan pig populations. Again, Sichuan and Yunnan Tibetan populations together with Neijiang and Rongchang defined a subgroup separating from the Tibet populations. These findings collectively support the contribution of geographically neighboring lowland populations to the today’s Tibetan populations. The historical admixture events, at least to a certain extent, are responsible for the substantial inter-population differentiation in Tibetan pigs. Altogether, we believe that Tibetan pig populations have experienced distinct demographic histories and have various degree of admixture with different neighboring populations. In general, Tibetan pigs can be divided into 3 independent populations: Tibet, Gansu and Sichuan & Yunnan.

### Distinct selection-nominated loci in different Tibetan pig populations

In Tibetans, a list of genes has been highlighted as potential targets of natural selection (reviewed in [12]). While *EPAS1* and *EGLN1* at the top list are consistently identified in multiple Tibetan studies, many of these selection candidate genes are unique to each study. Such differences may be at least partly attributed to different demographic histories and genetic differentiation among Tibetan populations that likely have various degree of admixture with different neighboring populations [23, 26, 27]. Tibetan pigs show similar population history patterns to their owners. As mentioned above, our genetic data suggest long-term population isolation and genetic differentiation among Tibetan pig populations with potential for admixture with geographic neighbors. It is therefore reasonable to speculate that each of Tibetan pig population may have unique adaptive variants. Our genome scans on individual population confirm the speculation. The SNP outliers in one population are largely different from the others (Figure 4). Especially, the top SNPs corresponding to *C9ORF3*, *GRIN2B* and *GRID1* genes exhibit population specific signals in the Gansu, Tibet and Sichuan & Yunan populations, respectively (Figure 4). These candidate genes are worthy mentioning because of their statistical significance and function implications. Further investigations of their potential role in the high-altitude adaptation of Tibetan pigs are worthwhile.

*C9ORF3*, also known as *Aminopeptidase O*, encodes a member of the M1 zinc aminopeptidase family that catalyzes the hydrolysis of amino acid residues from the N-terminus of peptide. C9ORF3 is involved in the renin-angiotensin pathway, in which C9ORF3 cleaves angiotensin III to generate angiotensin IV [28]. It is known that angiotensin IV regulates the vasoconstriction [29, 30] and the hydromineral balance as well as arterial blood pressure [31]. Moreover, high expression level of *C9ORF3* is positively correlated with maximal oxygen uptake and the percentage of type 1 fibers in humans [32]. As predicted by MalaCards [33], *C9ORF3* is associated with newborn respiratory distress syndrome. Therefore, preferential selection on the *C9ORF3* gene is likely required for Tibetan (Gansu) pigs to increase oxygen uptake and avoid pulmonary or cerebral vascular hypertension and edema during the long-standing living in the hypoxic highland.

*GRIN2B* encodes a subunit of N-methyl-D-apartate receptor that is the predominant excitatory neurotransmitter receptor in the mammalian brain. The receptor has a central role in memory and cognitive function. Defects in the *GRIN2B* gene have been found in human patients with mental retardation [34]. *GRID1* encodes glutamate receptor delta 1, a subunit of glutamate receptor channels that mediate most of the fast excitatory synaptic transmission in the central nervous system and play key roles in synaptic plasticity [35]. Variations in the promoter region of the *GRID1* gene have been associated with human schizophrenia [36, 37]. Positive selection of these genes may suggest the importance of neural regulation in the establishment of quickly response to attack under grazing conditions in Tibetan pigs.

### Common selection targets in Tibetan pigs

In addition to population-specific candidate loci, we highlighted a list of selection-nominated candidate genes shared by all Tibetan pigs. Many of these genes are functionally related to energy metabolism, angiogenesis, melanin synthesis and behavior defense response. Of note, *PLA2G12A* and *RGCC* in our top-ranking list stand out as strong candidate genes that likely play a role in high-altitude adaptation in Tibetan pigs.

*PLA2G12A* encodes secreted phospholipase A2, group XIIA enzyme. The enzyme hydrolyzes phospholipids into arachidonic acid and other lipophilic molecules that exert a variety of biological effects. Of note, arachidonic acid plays an important role in regulation of pulmonary vascular tone in lungs of both newborns and adults. In pigs and other species, arachidonic acid can induce dilation in pulmonary arteries. Altered arachidonic acid metabolites have been implicated in the development of pulmonary hypertension in chronically hypoxic piglets [38]. Therefore, *PLA2G12A* is a strong candidate gene contributing to high-altitude adaptation in Tibetan pigs. High frequencies of *PLA2G12A* favorable mutations in Tibetan pigs could be beneficial for increasing blood flow at low degree of hypoxic pulmonary hypertension.

*RGCC* plays a critical role in hypoxia-induced antiangiogenesis. It has a physical interaction with *HIF1*α and vascular endothelial growth factor (*VEGF*) that are key mediators in cellular response to hypoxia and ischemia [39]. In response to acute hypoxia, *HIF1*α activates the expression of *VEGF* that is critically important for skeletal muscle angiogenesis. The increased growth of blood vessels has been associated with capillary leak [40]. Therefore, the preferentially selected variants in the *RGCC* gene may inhibit the capillary growth and consequently avoid capillary leak in Tibetan pigs under long-term exposure to the hypoxic environment.

*EPAS1*, the “master regulator” of erythropoiesis in the HIF pathway (reviewed in [41]), also appear to be a target of selection in this study. *EPAS1* allelic variants that are common in Tibetan pigs may be loss of function and be negative associated with hemoglobin concentrations as a blunted erythropoietic response observed in Tibetans and Tibetan pigs [1–3]. It should be noted that the other well-characterized hypoxia gene *EGLN1* is not a target of selection in Tibetan pigs.

### A complex picture of the genetic mechanism underlying high-altitude adaptation in Tibetan pigs

In this study, we identified a total of 489 genes as potential selection targets in all Tibetan pig populations (Additional file 6: Table S2). A majority of these candidate genes have not been implicated in previous genome-wide studies on highlanders and high-altitude animals. Many genes are functionally related to high-altitude physiology, such as angiogenesis (*RGCC*), pulmonary hypertension remodeling (*PLA2G12A*), oxygen intake (*C9ORF3*), neural response (*GRID1* and *GRIN2B*), melanin synthesis (*MITF*, *PDGFRB* and *PIK3R3*) and heart development (*FOXS1*, *GAA* and *MYLK2*). Further characterization of these genes will be necessary to identify variants responsible for the observed signals. Both distinct and shared genomic signatures of selection were illustrated in Tibetan regional pig populations. We argue that both independent and shared adaptive variants could be responsible for local adaptation in each Tibetan pig population. Our findings reflect a complex picture of the genetic mechanism underlying high-altitude adaptation in Tibetan pigs. The adaptive mechanism seems to involve a range of genes regulating diverse biological processes. Distinct set of genes including some broadly shared loci act in a highly coordinated manner to offset severe physiological challenges imposed by the harsh hypoxic environment in different Tibetan pig populations.

### Future analyses

Our analysis provides a list of potential genes involved in high-altitude adaptation in Tibetan pigs, which will be the ground for future investigations. It is worthwhile to conduct resequencing of the most promising genomic regions. Haplotype similarity analysis based on the resequencing-called variants would allow us to identify Tibetan specific and minimal-shared haplotypes. The functional variants residing in these haplotypes, such as protein-altering mutations and regulatory mutations at the evolutionary conserved sites, would be strong candidate causative mutations.

Although we identified a list of candidate genes for high-altitude adaptation in Tibetan pigs, we are limited by the ascertainment bias and low genomic coverage of our SNP dataset. The 60 K SNPs on the Illumina porcine DNA chip were primarily characterized from Western pigs that have relatively higher LD extents compared to Tibetan pigs [19]. A mass of Tibetan specific variants are not included in the chip. Therefore, we must not have the power to identify all variants under positive selection for local adaptation of Tibetan pigs. Own to the substantially decreasing cost, whole-genome sequencing is now affordable in domestic animals. Such population-scale genome analyses on a broad sample of representative individuals would identify additional adaptive variants and further improve our understanding of the genetic basis of high-altitude adaptation in Tibetan pigs.

## Conclusions

Tibetan pig populations have experienced substantial genetic differentiation. Geographically neighboring lowland breeds likely contributed to Tibetan pigs by historical admixture events. The present-day Tibetan pigs can be divided into 3 independent populations: Tibet, Gansu and Sichuan & Yunnan. After a long period of colonization in the Qinghai-Tibetan Plateau, Tibetan pigs have developed a complex biological adaptation mechanism that could be different from that of Tibetans and other highland animals. Different Tibetan pig populations appear to have both distinct and convergent adaptive loci for the harsh environment of the Plateau.

## Methods

### Ethics statement

All animal work was conducted according to the guidelines for the care and use of experimental animals established by the Ministry of Agriculture of China. The Ethics Committee of Jiangxi Agricultural University specifically approved this study.

### Animals

A total of 678 pigs from 5 Tibetan geographic populations and 34 Chinese and Western lowland breeds were investigated in this study. These pigs are unrelated animals with no common ancestry for 3 generations. Boars were preferentially collected to cover consanguinity as broadly as possible. Of the 678 pigs, 304 individuals from 18 breeds have been tested in our previous study [19], and SNP data of 85 pigs from 6 breeds were retrieved from the Dryad Digital Repository: http://dx.doi.org/105061/dryad.t1r3d [42]. Sample size and origin of each Tibetan population and lowland breeds are given in Additional file 1: Table S1, and the geographic locations of 33 Chinese breeds including the 5 Tibetan populations are shown in Figure 1. Genomic DNA was extracted from ear tissues using a routine phenol/chloroform protocol, and was diluted to a final concentration of 20 ng/ml.

### SNP genotyping

All animals were genotyped for ~62,000 SNPs on Porcine SNP60 BeadChips [43] (Illumina, USA) according to the manufacturer protocol. SNP genotypes were recorded using BEADSTUDIO version 3.2 (Illumina, USA). SNPs were filtered with the criterion of call rate > 95% and minor allele frequency (MAF) > 0.05. A total of 41,495 informative SNPs were obtained, and different subsets of SNP data were chose from the 41,495 SNPs for further statistical analyses (see below). SNP genomic positions correspond to the current pig genome assembly (*Sscrofa*10.2).

A panel of 56 SNP markers at an average interval of 5.4 kb (Additional file 9: Table S3) covering 3 well-characterized hypoxia genes (*EGLN*, *EPAS1* and *ADAM1*) was genotyped on 324 Chinese indigenous pigs by the iPLEX MassARRAY platform (Sequenom, USA) according to the supplier’s protocol. SNP genotype calls were filtered with MAF > 0.05 and genotype call rate > 95%, resulting in 49 informative SNPs. The 49 SNPs were merged into the illumina SNPs to form a common subset of 41,544 SNP data in Chinese indigenous pigs, which were then used to detect signals of selection on the 3 hypoxia genes in Tibetan pigs.

### Population genetic and structure analyses

A common subset of 25,340 SNPs with MAF ≥ 0.2 in all tested pigs were explored to calculate three measures of genetic variability of each population: allelic richness (A_R_), the proportion of polymorphic markers (P_N_) and expected heterozygosity (H_E_) using ADZE [44] and PLINK [45] as described previously [19]. All 41,495 informative SNPs were used to analyze the pairwise genetic distance between populations as shown in our previous study [19]. Neighbor joining relationship trees between individuals were constructed using Neighbor in the PHYLIP version 3.69 package [46] and visualized by Figtree v1.4.0 (BEAST Software, http://beast.bio.ed.ac.uk/FigTree). A subset of 14,202 SNPs with low linkage disequilibrium (*r*^2^ < 0.3) filtered by PLINK [45] was employed to perform principal component analysis using the Smartpca program from EIGENSOFT [47].

The genetic differentiation between populations was assessed by the F_ST_ fixation index. Unbiased genetic differentiation estimates of F_ST_ were calculated as described in Reich’s paper [48] using the whole SNP dataset. Briefly, F_ST_ was estimated as follows:


Where


In the above formulae, n_i_ denotes the sample size in the ith population, and a_i_ is the counts of SNP allele A in the ith population (i = 1, 2). Because the range of F_ST_ is originally defined between 0 and 1 [49], negative F_ST_ values that do not have a biological interpretation were set to 0.

Population structure was analyzed using the Maximum Likelihood approach implemented in ADMIXTURE v 1.20 [50] and the above- mentioned 14,202 SNPs of low LD extents. The ADMIXTURE program was run in an unsupervised manner with a variable number of clusters (K = 2 to 8). The lowest 10-fold cross-validation values were used to choose an optimum K-value according to the default termination setting.

TreeMix [21] was employed to infer the patterns of population historical splits and mixtures for Tibetan populations in the context of diverse Chinese breeds. TreeMix estimates the historical relationships of sampled populations with a particular focus on topology rather than on the timing of demographic events. In the maximum likelihood trees, nodes represented inferred population splits, edges indicated the populations having ancestry from multiple parental populations, and branch lengths were proportional to the amount of genetic drift that populations have undergone. Migration events were modeled for populations that did not fit well the bifurcating tree model. These populations having ancestry from multiple parental populations were indicated as arrows. The color shades of the arrows reflected the relative weight of migration. Here, maximum-likelihood trees of Chinese pig populations without migration events and with 6 migration events were tested by using the Chinese wild boars as the outgroup population.

### Genome-wide tests of signatures of positive selection

LSBL statistics, a robust indicative of selection-nominated loci [51], were calculated for each polymorphic site of 41,495 informative SNPs under a three-group contrasting model. To identify population-shared signatures of selection, we treated Tibetan pigs as the highland group, and divided 20 Chinese lowland breeds into two lowland groups that were splited by TreeMix. Group 1 comprises 10 breeds including Hetao, Min, Laiwu, Jinhua, Jiangquhai, Erhualian, Meishan, Tongcheng, Ganxi and Shaziling pigs. Group 2 also consists of 10 breeds including Neijiang, Rongchang, Minguang, Diannan, Congjiang Xiang, Dahuabai, Bamaxiang, Dongshan, Luchuan and Wuzhishan pigs. To detect population-specific loci under selection, we treated one Tibetan population as the highland group, the remaining Tibetan populations as one contrasting group and the 20 Chinese lowland breeds as another contrasting group. To calculate LSBL values, we computed pairwise F_ST_ using the above Reich’s calculation at every SNP position for each two-way group comparison (i.e., Tibetan to Lowland group 1, Tibetan to Lowland group 2, and Lowland group 1 to Lowland group 2). Next, the pairwise F_ST_ values were used to calculate the LSBL at each SNP as described previously [48]. With three contrasting groups, SNPs showing Tibetan specific F_ST_ were identified as candidate selection loci. LSBL outliers were defined as sites with LSBL statistics surpassing 0.5% of the empirical distributions.

### Characterization of candidate genes under selection

The 50 kb upstream and downstream of significant LSBL loci were operationally defined as candidate regions under selection. Pig annotated genes within candidate regions were first searched against the pig genome assembly 10.2 [52] via the Ensembl Genome Browser (http://ensembl.org/index.html). Furthermore, human orthologous genes were identified by aligning pig gene-associated regions against the human genome using the BLAST-like Alignment Tool [53]. To perform functional enrichment of the candidate genes, the human Gene Ontology database (http://www.geneontology.org) was then queried by the ClueGO plugin of Cytoscape [54] using Symbol ID as input parameters. The enriched GO terms and KEGG pathways were characterized according to the default setting.

### Data availability

The genotyping data set supporting the results of this article is available in the Dryad database (doi:10.5061/dryad.53j31).

## Electronic supplementary material

Additional file 1: Table S1: Summary of genetic diversity of each tested population. (DOC 110 KB)

Additional file 2: Figure S1: The neighbor-joining tree of all tested breeds and Tibetan pig populations based on genome-wide allele sharing. The tree illustrates a clear evolution split between Chinese and Western pigs. It also shows obvious genetic differentiation among Tibetan pig populations that usually group together with their geographic neighbors. Two Chinese synthetic breeds including Sutai and Lulai define intermediate branches between Chinese and Western groups. Such intermediate branches were also observed for Chinese Licha and Kele pigs, corresponding to our previous findings of the historical introgression of Western pigs into the two Chinese populations [19]. Tibet 1, the Tibetan pig from Gongbujiangda in the Tibet Autonomous Region. Tibet 2, the Tibetan pig from Milin in the Tibet Autonomous Region. (TIFF 5 MB)

Additional file 3: Figure S2: Principle components analysis of all tested breeds and Tibetan pig populations in the present study. Principal component (PC) 1 (x-axis) verus PC2 (y-axis). PC1 clearly discriminates Chinese and Western pigs. PC2 separates Chinese pigs including Tibetan populations in a manner corresponding to their geographic locations. The 5 Tibetan pig populations highlighted in shade exhibit genetic similarity to their geographic neighbors. The two synthetic breeds (Sutai and Lulai) and two admixed breeds (Licha and Kele) show consistent signals of admixture with Western pigs. (TIFF 339 KB)

Additional file 4: Figure S3: Population structure of each population revealed by the ADMIXTURE software. At K = 8, the ancestry structures of Tibetan pigs from Sichuan and Yunnan were nearly identical, differing from those of Tibetan pigs from Tibet and Gansu. From K = 2 to K = 5, ~80% of the Western pig genomes were assigned to Western wild boars. Of note, about 20% of Chinese ancestry was consistently observed in Landrace and Large White, suggesting a historical admixture between Chinese and Western pigs. The observation is in agreement with the previous report of a ~35% Asian fraction in Western pigs according to the whole genome sequence data [52]. The abbreviation of each breed is identical to that given in the legend of Figure 1. (TIFF 12 MB)

Additional file 5: Figure S4: TreeMix plot for residual fit from the maximum likelihood tree without migration events. (TIFF 738 KB)

Additional file 6: Table S2: SNP outliers and candidate genes for high-altitude adaptation in each and all geographic populations of Tibetan pigs. (XLSX 132 KB)

Additional file 7: Figure S5: A venn diagram showing shared and distinct candidate genes in each and all geographic populations of Tibetan pigs. Numbers indicating how many genes belong to each of Tibetan populations are shown in the graph. (TIFF 488 KB)

Additional file 8: Figure S6: Patterns of selection signatures within three well-characterized hypoxia genes in Tibetan pigs. (A) Distribution of LSBL values within the target regions. LSBL values are plotted along the y-axis, and the threshold indicating signature of selection is denoted with a dashed grey line. The candidate gene (*EPAS1*, *EGLN1* and *ADAM17*) names and their corresponding regions are indicated below each panel. (B) Allele frequencies of the two outlier SNPs at the *EPSA1* regions in a panel of Chinese indigenous pig populations. The breed codes are identical to those given in the legend of Figure 1. (TIFF 747 KB)

Additional file 9: Table S3: Genotypes of 56 SNP markers within 3 well- characterized hypoxia genes (*EGLN*, *EPAS1* and *ADAM1*) in 324 Chinese indigenous pigs. (XLSX 122 KB)

## Abbreviations

GO: Gene ontology
HIF: Hypoxia-inducible factor
KEGG: Kyoto encyclopedia of genes and genomes
LD: Linkage disequilibrium
LSBL: Locus-specific branch length
MAF: Minor allele frequency
NJ: Neighbor joining
PCA: Principal component analysis
SCYN: Sichuan & Yunnan
VEGF: Vascular endothelial growth factor
w: Migration weight.
