# Supplementary material for: Population history and genomic signatures for high-altitude adaptation in Tibetan pigs
Source: BMC Genomics. 2014 Oct 1;15(1):834. doi: 10.1186/1471-2164-15-834 (PMC4197311; doi:10.1186/1471-2164-15-834)
Supplement: Supplementary file 1 — Additional file 1: Table S1: Summary of genetic diversity of each tested population. (DOC 110 KB) [file 12864_2014_6536_MOESM1_ESM.doc]

**Table S1.** Summary of genetic diversity of each tested population ^a^

| Breed | | Sample Size | Origin (county, province) | Altitude (m) | N_SNP_ | A_R_ | P_N_ | H_E_ |
| --- | --- | --- | --- | --- | --- | --- | --- | --- |
| Chinese pigs | |  |  |  |  |  |  |  |
| 1 | Bama Xiang | 16 | Bama, Guangxi | 830 | 12676 | 1.81 | 0.83 | 0.28 |
| 2 | Bamei | 16 | Huangzhong, Qinghai | 2400 | 13740 | 1.87 | 0.88 | 0.31 |
| 3 | Congjiang Xiang | 16 | Congjiang, Guizhou | 500 | 10917 | 1.68 | 0.69 | 0.24 |
| 4 | Dahuabai | 16 | Dongguan, Guangdong | 40 | 11583 | 1.73 | 0.74 | 0.25 |
| 5 | Diannan small ear | 15 | Jinghong, Yunnan | 550 | 13061 | 1.82 | 0.83 | 0.28 |
| 6 | Dongshan | 15 | Quanzhou, Guangxi | 250 | 11301 | 1.69 | 0.70 | 0.24 |
| 7 | Erhualian | 32 | Wuxi, Jiangsu | 10 | 12399 | 1.88 | 0.89 | 0.28 |
| 8 | Ganxi | 13 | Shanggao, Jiangxi | 50 | 9435 | 1.61 | 0.62 | 0.22 |
| 9 | Hetao large ear | 16 | Wuyuan, Inner Mongolia | 1200 | 15608 | 1.91 | 0.92 | 0.33 |
| 10 | Jiangquhai ^b^ | 11 | Taizhou, Jiangsu | 10 | 11670 | 1.76 | 0.78 | 0.26 |
| 11 | Jinghua | 13 | Jinhua, Zhejiang | 50 | 8910 | 1.60 | 0.62 | 0.21 |
| 12 | Jinghua ^b^ | 17 | Jinhua, Zhejiang | 50 | 9933 | 1.62 | 0.63 | 0.21 |
| 13 | Kele | 10 | Hezhang, Guizhou | 1700 | 20099 | 1.97 | 0.98 | 0.38 |
| 14 | Laiwu | 18 | Laiwu, Shandong | 250 | 12639 | 1.86 | 0.87 | 0.29 |
| 15 | Licha | 14 | Qingdao, Shandong | 50 | 19678 | 1.98 | 0.98 | 0.39 |
| 16 | Luchuan | 18 | Luchan, Guangxi | 70 | 10051 | 1.69 | 0.70 | 0.23 |
| 17 | Lulai | 3 | Laiwu, Shandong | 250 | 14652 | 1.84 | 0.77 | 0.34 |
| 18 | Meishan ^b^ | 17 | Jiading, Shanghai | 5 | 11869 | 1.76 | 0.77 | 0.26 |
| 19 | Min | 22 | Lanxi, Heilongjiang | 240 | 15773 | 1.87 | 0.87 | 0.32 |
| 20 | Mingguang small ear | 16 | Tengcong, Yunnan | 1600 | 16072 | 1.96 | 0.96 | 0.35 |
| 21 | Neijiang | 16 | Neijiang, Sichuan | 350 | 11896 | 1.85 | 0.87 | 0.27 |
| 22 | Rongchang | 18 | Rongchang, Chongqin | 350 | 12738 | 1.83 | 0.84 | 0.29 |
| 23 | Shaziling | 11 | Xiangtan, Hunan | 100 | 12213 | 1.73 | 0.74 | 0.27 |
| 24 | Sutai | 15 | Suzhou, Jiangsu | 5 | 18324 | 1.95 | 0.96 | 0.36 |
| 25 | Tibetan | 21 | Hezuo, Gansu | 3100 | 13033 | 1.86 | 0.87 | 0.29 |
| 26 | Tibetan | 29 | Gongbujiangda, Tibet | 3600 | 13694 | 1.88 | 0.89 | 0.30 |
| 27 | Tibetan | 16 | Milin, Tibet | 3600 | 16277 | 1.97 | 0.98 | 0.35 |
| 28 | Tibetan | 16 | Litan, Sichuan | 4000 | 13285 | 1.81 | 0.82 | 0.29 |
| 29 | Tibetan | 19 | Diqing, Yunnan | 3300 | 14525 | 1.89 | 0.91 | 0.31 |
| 30 | Tongcheng | 16 | Tongcheng, Hubei | 100 | 13573 | 1.89 | 0.91 | 0.30 |
| 31 | Wuzhishan | 16 | Qiongshan, Hainan | 330 | 14666 | 1.90 | 0.91 | 0.32 |
| 32 | Xiang ^b^ | 13 | -- | -- | 10847 | 1.72 | 0.73 | 0.25 |
| 33 | Chinese Wild Boar | 15 | Nanchang, Jiangxi | 150 | 15161 | 1.89 | 0.89 | 0.32 |
|  |  | 4 | Shangyou, Jiangxi | 150 |  |  |  |  |
|  |  | 2 | Ningbo, Zhejiang | 100 |  |  |  |  |
| Western pigs | |  |  |  |  |  |  |  |
| 34 | Duroc | 35 | USA | \ | 12692 | 1.84 | 0.85 | 0.28 |
| 35 | Landrace | 35 | Danmark | \ | 15624 | 1.94 | 0.95 | 0.33 |
| 36 | Large White | 35 | Canada | \ | 16613 | 1.96 | 0.96 | 0.34 |
| 37 | White Duroc | 5 | USA | \ | 15978 | 1.76 | 0.76 | 0.29 |
| 38 | Hampshire ^b^ | 14 | USA | \ | 10861 | 1.73 | 0.74 | 0.24 |
| 39 | Western Wild Boar ^b^ | 13 | -- | \ | 9811 | 1.60 | 0.61 | 0.22 |

^a^N_SNP_, the number of SNPs with MAF >0.2 in the 25,340 SNP subset; P_N_, the proportion of SNP which displayed polymorphism in the 25,340 SNPs selected from the 60 K panel; A_R_, allelic richness; H_E_, expected heterozygosity.

^b^ Data was retrieved from the Dryad database (http://datadryad.org/).
