# Supplementary figures and images for: Population history and genomic signatures for high-altitude adaptation in Tibetan pigs
Source: BMC Genomics. 2014 Oct 1;15(1):834. doi: 10.1186/1471-2164-15-834 (PMC4197311; doi:10.1186/1471-2164-15-834)

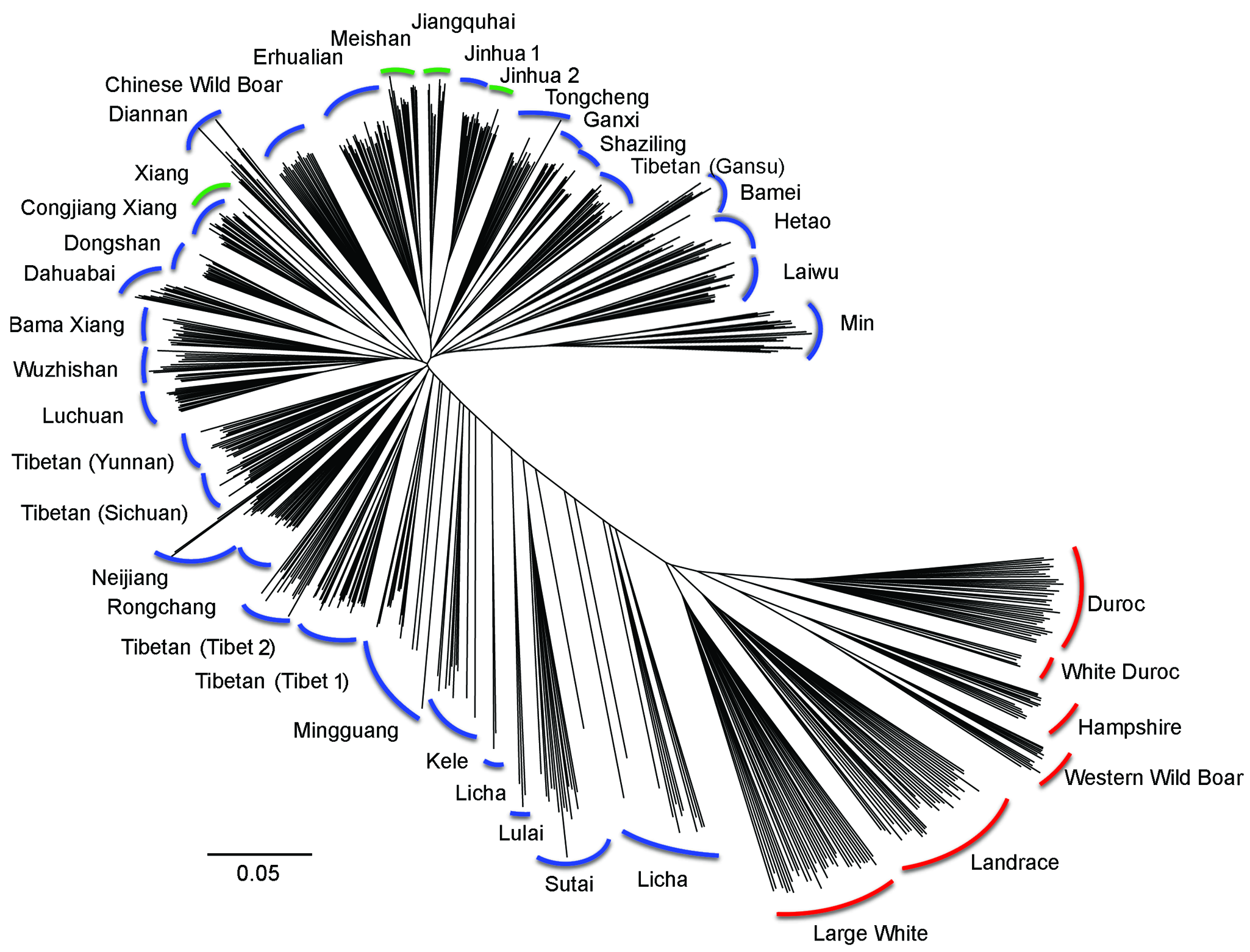

Supplement: Supplementary file 2 — Additional file 2: Figure S1: The neighbor-joining tree of all tested breeds and Tibetan pig populations based on genome-wide allele sharing. The tree illustrates a clear evolution split between Chinese and Western pigs. It also shows obvious genetic differentiation among Tibetan pig populations that usually group together with their geographic neighbors. Two Chinese synthetic breeds including Sutai and Lulai define intermediate branches between Chinese and Western groups. Such intermediate branches were also observed for Chinese Licha and Kele pigs, corresponding to our previous findings of the historical introgression of Western pigs into the two Chinese populations [19]. Tibet 1, the Tibetan pig from Gongbujiangda in the Tibet Autonomous Region. Tibet 2, the Tibetan pig from Milin in the Tibet Autonomous Region. (TIFF 5 MB) [file 12864_2014_6536_MOESM2_ESM.tiff]

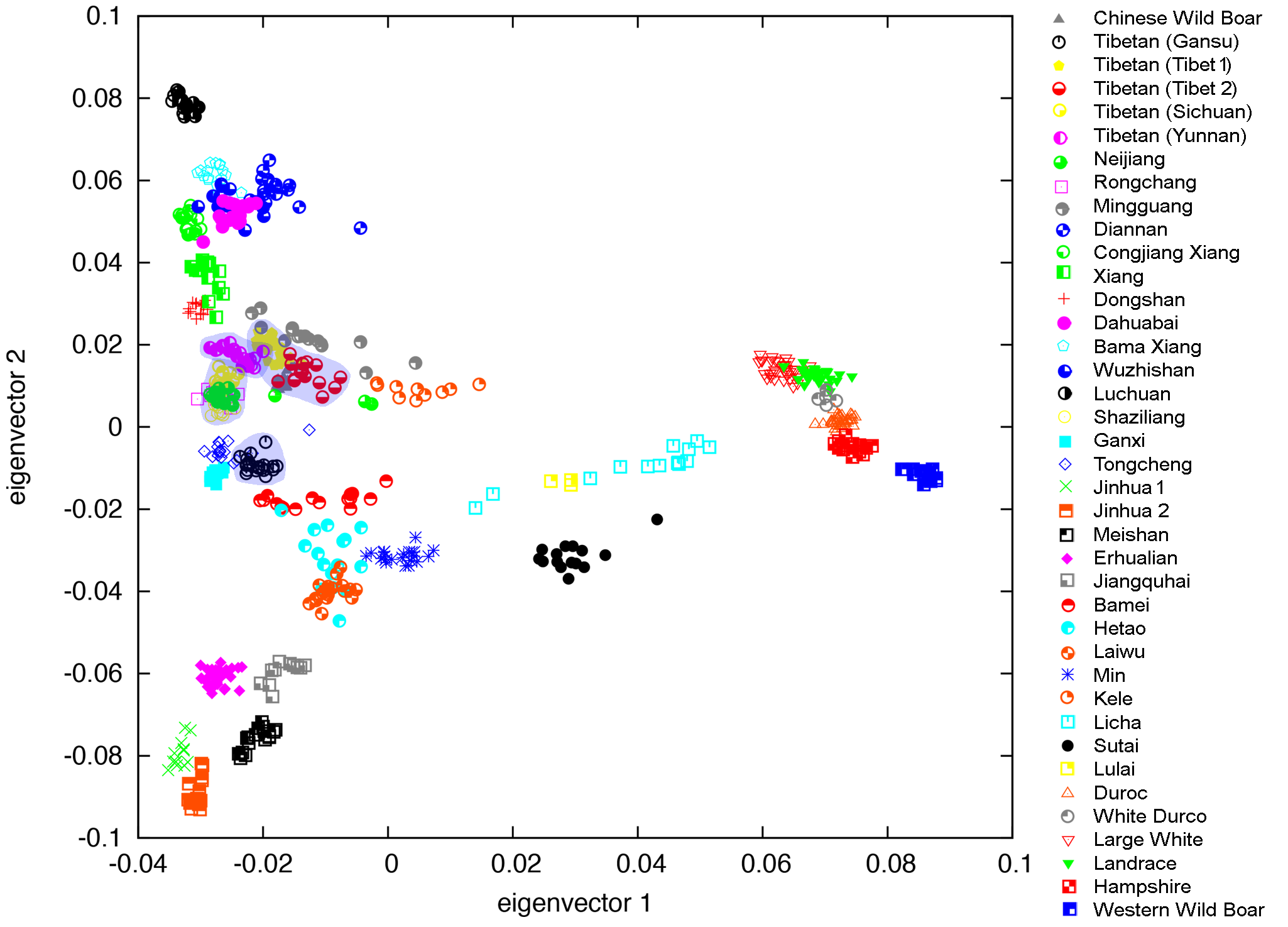

Supplement: Supplementary file 3 — Additional file 3: Figure S2: Principle components analysis of all tested breeds and Tibetan pig populations in the present study. Principal component (PC) 1 (x-axis) verus PC2 (y-axis). PC1 clearly discriminates Chinese and Western pigs. PC2 separates Chinese pigs including Tibetan populations in a manner corresponding to their geographic locations. The 5 Tibetan pig populations highlighted in shade exhibit genetic similarity to their geographic neighbors. The two synthetic breeds (Sutai and Lulai) and two admixed breeds (Licha and Kele) show consistent signals of admixture with Western pigs. (TIFF 339 KB) [file 12864_2014_6536_MOESM3_ESM.tiff]

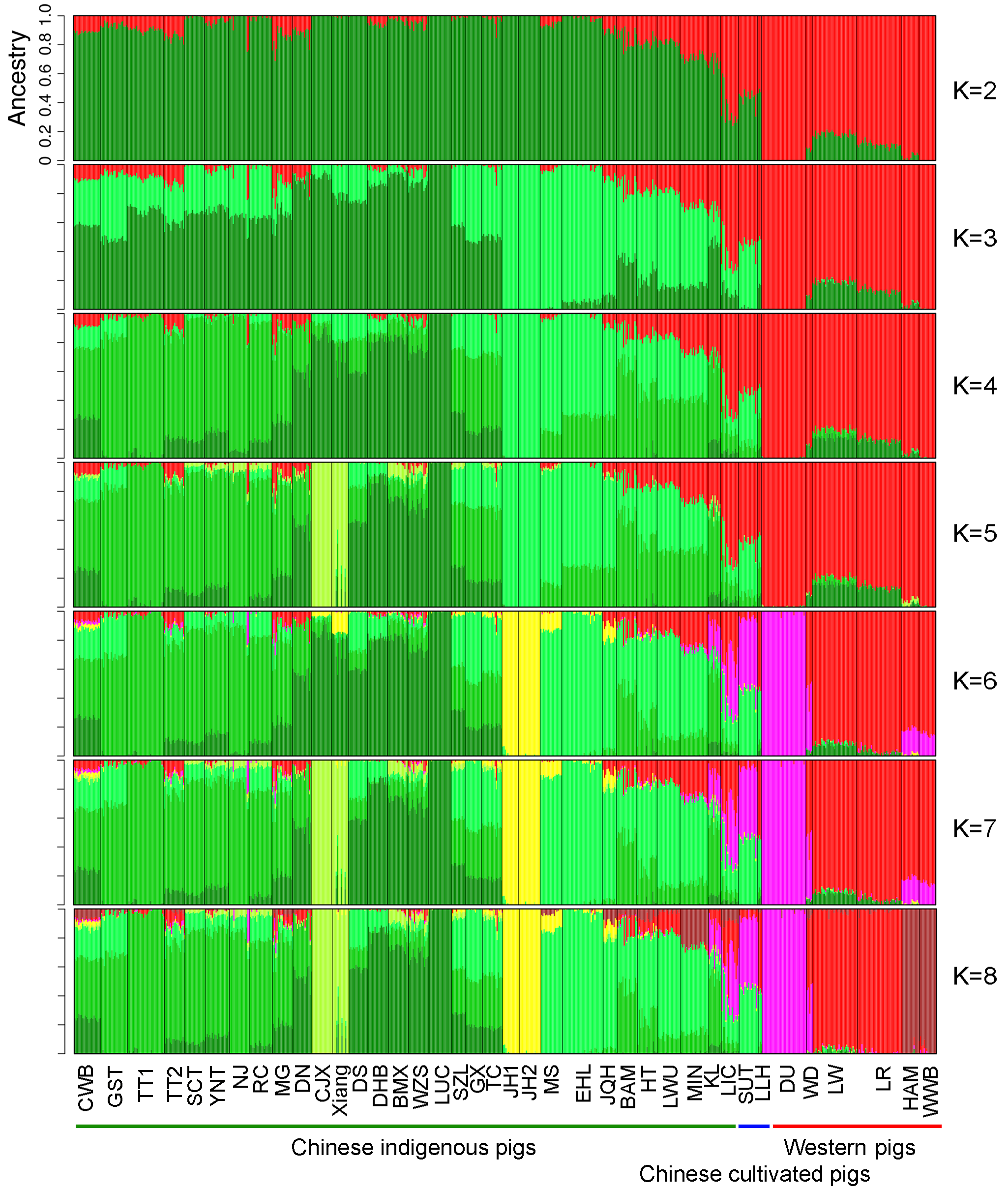

Supplement: Supplementary file 4 — Additional file 4: Figure S3: Population structure of each population revealed by the ADMIXTURE software. At K = 8, the ancestry structures of Tibetan pigs from Sichuan and Yunnan were nearly identical, differing from those of Tibetan pigs from Tibet and Gansu. From K = 2 to K = 5, ~80% of the Western pig genomes were assigned to Western wild boars. Of note, about 20% of Chinese ancestry was consistently observed in Landrace and Large White, suggesting a historical admixture between Chinese and Western pigs. The observation is in agreement with the previous report of a ~35% Asian fraction in Western pigs according to the whole genome sequence data [52]. The abbreviation of each breed is identical to that given in the legend of Figure 1. (TIFF 12 MB) [file 12864_2014_6536_MOESM4_ESM.tiff]

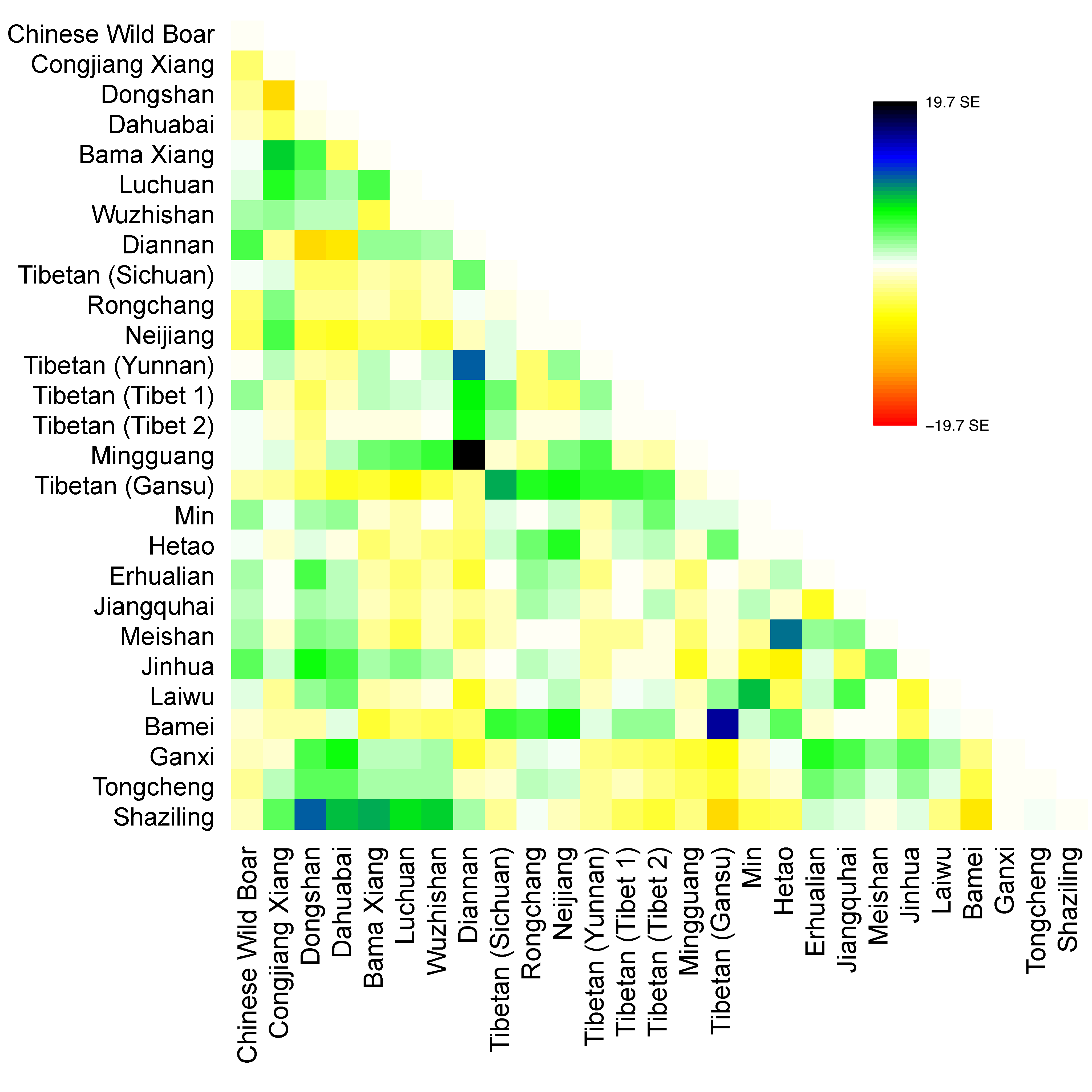

Supplement: Supplementary file 5 — Additional file 5: Figure S4: TreeMix plot for residual fit from the maximum likelihood tree without migration events. (TIFF 738 KB) [file 12864_2014_6536_MOESM5_ESM.tiff]

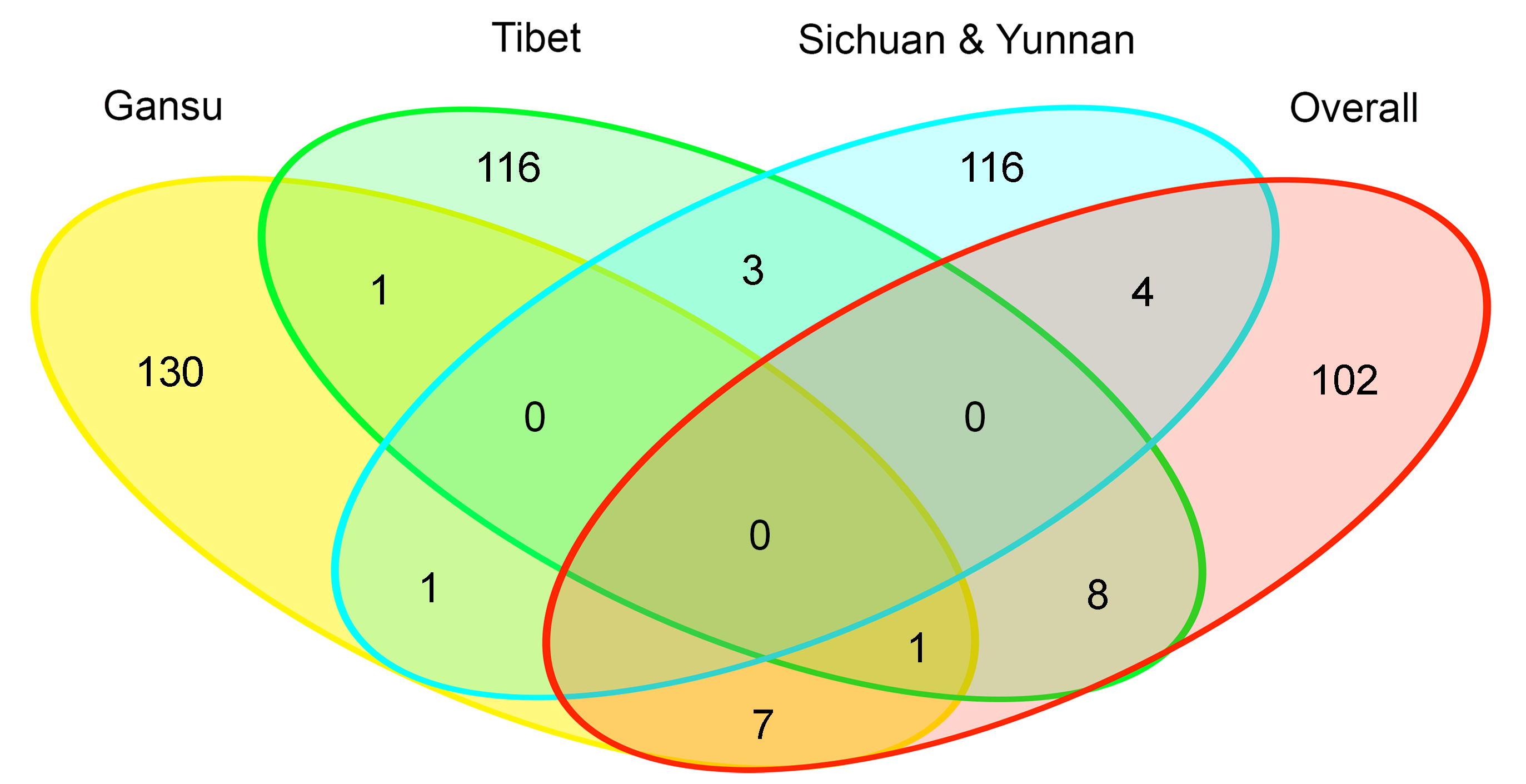

Supplement: Supplementary file 7 — Additional file 7: Figure S5: A venn diagram showing shared and distinct candidate genes in each and all geographic populations of Tibetan pigs. Numbers indicating how many genes belong to each of Tibetan populations are shown in the graph. (TIFF 488 KB) [file 12864_2014_6536_MOESM7_ESM.tiff]

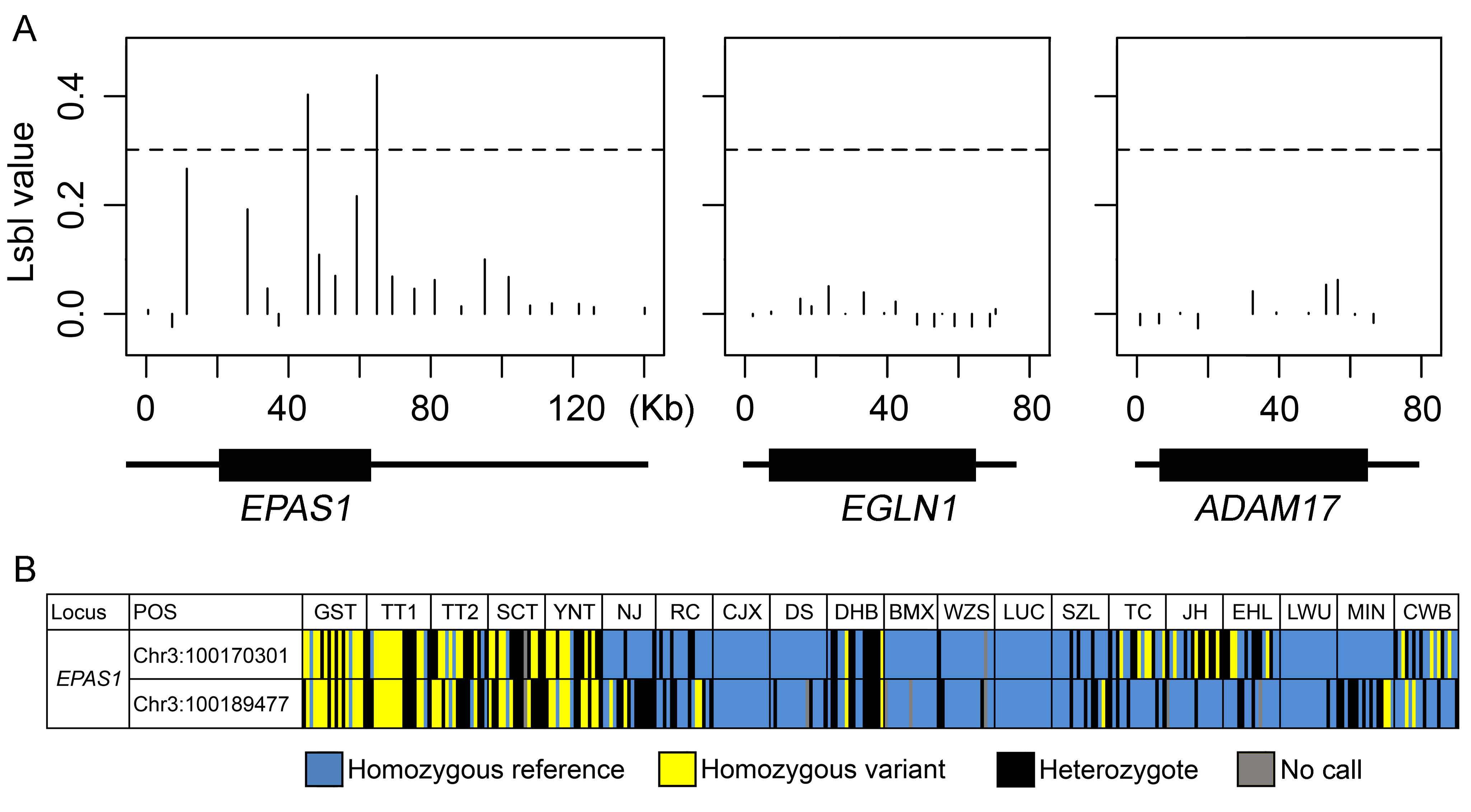

Supplement: Supplementary file 8 — Additional file 8: Figure S6: Patterns of selection signatures within three well-characterized hypoxia genes in Tibetan pigs. (A) Distribution of LSBL values within the target regions. LSBL values are plotted along the y-axis, and the threshold indicating signature of selection is denoted with a dashed grey line. The candidate gene (EPAS1, EGLN1 and ADAM17) names and their corresponding regions are indicated below each panel. (B) Allele frequencies of the two outlier SNPs at the EPSA1 regions in a panel of Chinese indigenous pig populations. The breed codes are identical to those given in the legend of Figure 1. (TIFF 747 KB) [file 12864_2014_6536_MOESM8_ESM.tiff]
